# Study on the Properties of Nano-CeO_2_/Polyurea-Based Gel Grease for Electric Motor Bearings

**DOI:** 10.3390/gels12060528

**Published:** 2026-06-12

**Authors:** Han Peng, Zihao Meng, Minzhang Zhao, Linjian Shangguan, Bing Li, Budi Peng, Yihao Zhang

**Affiliations:** 1 School of Mechanical Engineering, North China University of Water Resources and Electric Power, Zhengzhou 450045, China; mzh000311@163.com (Z.M.); david19881007@163.com (B.L.); pbd286500@163.com (B.P.); 18539131830@163.com (Y.Z.); 2 School of Water Conservancy, North China University of Water Resources and Electric Power, Zhengzhou 450045, China; 3 Zhengzhou Aote Technology Co., Zhengzhou 450045, China; zmz@autol.ne

**Keywords:** polyurea-based gel grease, nano-cerium dioxide, composite gel grease, physical adsorption, tribological properties

## Abstract

In response to the harsh operating conditions of high-speed, high-temperature bearings in new energy vehicle drive motors, this study focuses on enhancing the performance of polyurea-based gel greases through the use of nano-additives. Using polyurea-based gel grease as the matrix, nano-composite gel greases with different CeO_2_ loadings were prepared, and their tribological properties and rheological behavior were characterized using four-ball friction tests, rheological testing, and SEM analysis. The results indicate that adding 0.5 wt% CeO_2_ increased the grease’s yield stress by approximately 75.4% and significantly raised its apparent viscosity. This rheological enhancement effect may be attributed to the physical adsorption of ultrafine particles onto the polyurea fiber network and potential interfacial interactions between the particles and the fiber surfaces. Compared to the original grease, the average coefficient of friction decreased by 10.4%, and the average wear scar diameter decreased by 12.7%. Meanwhile, the shear stress increased by 110.41 Pa, and the viscosity increased by 1102 Pa·s. This study provides experimental evidence and technical references for the development of high-performance gel lubricants suitable for motor bearings operating under high-temperature conditions.

## 1. Introduction

As the global energy crisis and environmental pollution become increasingly severe, the development of pure electric vehicles has become a core strategy for sustainable development in the global transportation sector [1,2,3]. As the “heart” of a pure electric vehicle, the performance of the drive motor directly determines the vehicle’s power, efficiency, and reliability [4,5,6,7]. Compared to traditional industrial motors, drive motors in pure electric vehicles face severe challenges posed by higher power density, a wider speed range, and complex operating conditions such as frequent start–stops and high-load speed changes [8,9,10]. Against this backdrop, the operational status of the internal bearings—as key components that support the rotor system and transmit power—is of critical importance [11]. Grease, serving as the “lifeblood” of the bearings, plays a central role; its performance directly influences bearing friction, wear, temperature rise, and ultimately the energy efficiency and service life of the entire drive motor [12].

An ideal lubricant for drive motor bearings must maintain structural stability under high shear rates, retain appropriate consistency across a wide temperature range, and exhibit excellent anti-wear properties to withstand sudden impact loads [13,14]. Polyurea-based gel greases are considered one of the ideal lubricants for drive motor bearings in pure electric vehicles due to their outstanding high-temperature stability, oxidation stability, and mechanical stability [15]. However, the performance of conventional gel greases has approached their inherent physicochemical limits under increasingly severe operating conditions [16]. Therefore, the addition of functional additives to synergistically enhance their overall performance has become a hot research topic in the field of lubrication. Wu et al. [17] developed a composite calcium sulfonate gel-based grease using environmentally friendly materials, incorporating hexagonal boron nitride (hBN) and nano-Al_2_O_3_ as additives. Optimal friction-reducing, anti-wear, and vibration-damping properties were achieved when 0.75% hBN and 0.25% Al_2_O_3_ were added. Wu et al. [18] investigated the addition of 1% nano-CuO to three common greases (composite lithium-based, polyurea, and composite calcium sulfonate). The results indicated that nano-CuO had the most significant improvement effect on polyurea grease (PG), most effectively reducing friction, minimizing wear, and suppressing bearing vibration. Bartolome et al. [19] used two phosphorus-derived ionic liquids (IL1 and IL2) as additives, incorporating them into a lithium complex grease (G1) and an anhydrous calcium-based grease (G2), respectively, to evaluate their friction, wear, and oxidation resistance properties. The results indicated that IL2 significantly improved the friction-reducing properties of G1; both ionic liquids enhanced the wear resistance of G2; IL2 effectively improved G2’s resistance to oxidation and thermal aging. Bond et al. [20] investigated the effects of additives, such as silver (Ag) nanoparticles, on electrochemical surface pitting of steel contacts in polyurea-based gel greases. The results indicated that electrochemical pitting primarily occurred at points where the direction of motion changed and was accompanied by grease decomposition. The silver nanoparticle additive significantly suppressed pitting, while other additives exhibited similar but weaker effects. Prasad et al. [21] investigated the effects of mixing multi-walled carbon nanotubes (MWCNTs) as additives with lithium-based gel-like grease on the tribological properties and dynamic behavior of roller bearings. The addition of MWCNTs significantly increased the lubricant film stiffness and the roughness-to-load ratio, effectively reduced bearing vibration levels, and improved the overall performance of the bearings. Li et al. [22] developed a perfluoropolyether grease for electric motor bearings, which exhibited excellent overall performance: a wear scar diameter of only 0.41 mm, a sintering load as high as 7846 N, and a maximum non-seizing load of 1961 N. Zhao et al. [23] conducted a 518-h in-service performance evaluation of a long-life anti-wear motor bearing grease. The results showed that the used grease remained stable in terms of chemical structure, oxidation resistance, and anti-wear performance compared to the unused new product, confirming that this grease possesses excellent long-life characteristics and service stability. Wang et al. [24] outlined key points for routine maintenance of motor bearings and grease filling procedures, emphasizing the critical role of scientific lubrication in reducing bearing temperature rise and extending service life. By standardizing lubrication cycle selection, quantitative filling methods, and cleaning procedures, bearing failures caused by insufficient or excessive lubrication can be effectively prevented. Liang [25] effectively controlled the failure rate in temperature rise tests of traction motor bearings for high-speed trains by regulating the grease filling volume at both ends of the traction motor and improving the cleanliness of the bearings and bearing housings. Wang et al. [26] developed a new type of gel-like grease specifically for the bearings of high-speed train traction motors. All performance metrics of this grease met technical requirements. During bench tests simulating 120,000 km of operational mileage, the bearings maintained low temperatures and low vibration levels throughout, without requiring grease replenishment, confirming its excellent long-term lubrication performance and full compliance with the operational demands of high-speed trains. Peng et al. [27] used a gel-state Mobil SHC 461WT grease as the test material and employed a rheometer to study the effects of three additives (RFM3000, SK3115, and PV611) on its rheological properties. Combining the results with the H-B model, they found that RFM3000 provided the best improvement. Peng et al. [28] investigated the effects of blending polysiloxane viscosity modifier (PV611) with dialkyldithiocarbamate of molybdenum (RFM3000) at different ratios (3:1, 1:1, 1:3) on the tribological and rheological properties of Schaeffler Load 460 gel grease. The results showed that under the three mixing ratios, the coefficient of friction decreased by 57.2–71.9%, the wear scar diameter decreased by 44.5–61.3%, and the shear stress and viscosity increased by 117.94 Pa and 1295.02 mPa·s, respectively. Liu [29] successfully developed a base oil meeting the required specifications using naphthenic lubricating oil as the raw material through a blending process. Lu [30] independently developed a set of testing equipment for evaluating the service life of rolling bearing greases. By designing an axial loading mechanism and an auxiliary lubrication system, a comprehensive testing method and a service life reliability calculation model were established. The results indicate that temperature and rotational speed have a significant impact on grease service life, while the effect of grease evaporation on service life is negligible. Li [31] analyzed the influence of base oil, thickeners, and additives on the performance of gel-type greases. Based on NTN test standards, he designed test protocols for rapid acceleration, temperature rise, and durability, and successfully established a grease performance evaluation system that simulates actual operating conditions.

Nanomaterials show great potential as lubricant additives [32,33]. Due to characteristics such as the size-dependent effects and high surface activity, nano-additives can form an effective protective film on the surfaces of friction pairs or significantly improve the tribological properties of base greases through mechanisms such as the “micro-bearing” effect and self-healing. He et al. [34] added nano-CeO_2_ to lithium-based gel-like grease to investigate the effects of its content and temperature on friction and wear properties. The addition of 0.6 wt% nano-CeO_2_ yielded the best results, reducing the coefficient of friction and wear scar diameter by 28% and 13%, respectively. Gabriel et al. [35] compared the performance differences between lithium-based composite grease and polypropylene-based grease. After 337 h of long-term experimental evaluation, it was found that polypropylene-thickened grease performed superiorly in terms of friction torque and energy consumption, with energy consumption reduced by 21.5% compared to lithium-based composite grease. This confirmed that polypropylene-based grease exhibits superior energy efficiency and durability. Calderon et al. [36] found that the oil release characteristics of grease dominate the evolution of friction torque, torque hysteresis, and rheological changes; among these, polypropylene-based grease demonstrated the best energy consumption characteristics due to its superior oil release capability. Shah et al. [37] systematically summarized the latest research and development trends in electric vehicle lubricants and greases, pointing out that in-depth exploration of the mechanisms of action and performance optimization pathways for nano-additives is of critical importance for the development of next-generation high-performance electric vehicle greases. Gautam et al. [38] developed a novel gel-like grease co-thickened with lithium stearate and fumed silica, with the addition of sodium poly(4-styrenesulfonate). Tribological tests showed that its coefficient of friction was 65% lower than that of commercial greases, wear volume was reduced by a factor of 10, and surface roughness was reduced to 0.17 micrometers, while also providing better protection against copper corrosion. All performance indicators meet the stringent requirements of electric vehicles for grease conductivity, friction and wear resistance, and thermal stability.

Although research on nano-CeO_2_ as a grease additive has made some progress, systematic studies on its application in polyurea-based gel greases—particularly regarding the synergistic effects of its loading on the grease’s comprehensive performance and the underlying mechanisms—remain lacking [39]. Therefore, determining the optimal loading level of nano-cerium dioxide in polyurea-based gel lubricants holds significant theoretical guidance and engineering value for the development of next-generation high-performance gel lubricants specifically designed for electric vehicle drive motors. It should be noted that this study provides a preliminary evaluation of the tribological performance of this grease under moderate speed, moderate load, and 80 °C conditions, which can serve as a foundational reference for the development of grease for high-speed, high-temperature motor bearings [40]; however, its performance under actual high-speed, high-temperature operating conditions (>10,000 rpm, >120 °C) requires further research and validation.

This study aims to systematically investigate the reinforcing effect of nano-cerium oxide as a functional additive in polyurea-based gel grease, with a focus on analyzing the patterns of its influence on the grease’s comprehensive performance across a concentration gradient ranging from 0.1% to 2%. Polyurea-based gel greases, with their unique gel network structure, combine the high stability of gels with the excellent lubricating properties of greases; the introduction of nano-cerium oxide may exert a regulatory effect on this gel network structure. The study precisely quantified the long-term anti-wear performance of the grease through four-ball friction and wear tests, and used a rotational rheometer to thoroughly analyze the regulatory effect of nanoparticle introduction on the rheological properties of the polyurea-based gel grease system, thereby clarifying the impact of the nano-additive on the integrity of the gel network structure. The study elucidated the lubrication mechanism of the nano-additives under complex operating conditions and determined the optimal concentration for achieving synergistic improvements in tribological and rheological properties, thereby providing a theoretical basis for the formulation design and engineering application of a new generation of polyurea-based gel greases specifically tailored for the bearings of electric vehicle drive motors.

## 2. Results and Discussion

### 2.1. Tribological Properties Testing

**Figure 1:**
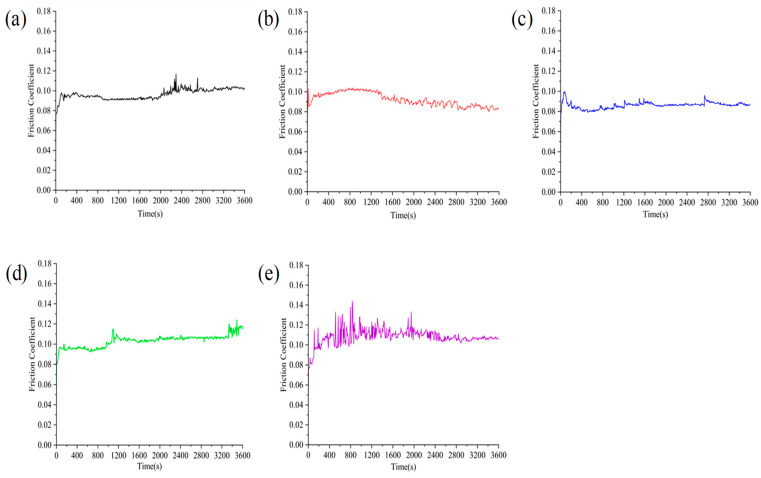
Friction coefficient versus time curves for polyurea-based gel greases with different CeO_2_ loadings. (**a**) Polyurea-based gel grease. (**b**) Polyurea-based gel grease + 0.1% CeO_2_. (**c**) Polyurea-based gel grease + 0.5% CeO_2_. (**d**) Polyurea-based gel grease + 1% CeO_2_. (**e**) Polyurea-based gel grease + 2% CeO_2_.

Figure 1 shows the variation in the friction coefficient of polyurea-based gel grease over time as a function of CeO_2_ loading.

As shown in Figure 1a, the coefficient of friction of the polyurea-based gel grease remained relatively stable from 920 s to approximately 1800 s, and began to fluctuate significantly at 2100 s. During the experiment, the minimum coefficient of friction was 0.084, the maximum was 0.117, and the average coefficient of friction was 0.096. Figure 1b shows the results of adding 0.1% mass fraction of cerium dioxide to the polyurea-based gel grease. The coefficient of friction remained relatively stable before 1300 s, but exhibited significant fluctuations after 1400 s. During the experiment, the minimum coefficient of friction was 0.08, the maximum was 0.104, and the average coefficient of friction was 0.092. As shown by the trend in Figure 1c, the friction coefficient of the polyurea-based gel grease containing 0.5% mass fraction of cerium dioxide remained stable throughout the experiment, and the total friction coefficient was relatively low. The friction coefficient reached a relatively stable state as early as 400 s. During the experimental process after the friction coefficient stabilized, the minimum value was 0.078, the maximum value was 0.1, and the average friction coefficient was 0.086. From the trend shown in Figure 1d, it can be observed that the coefficient of friction for the polyurea-based gel grease containing 1% mass fraction of cerium dioxide remained stable after 1300 s. After 3300 s, the coefficient of friction fluctuated significantly. During the experimental process after the coefficient of friction stabilized, the minimum value was 0.102, the maximum value was 0.124, and the average coefficient of friction was 0.103. As shown in Figure 1e, the friction coefficient of the polyurea-based gel grease containing 2% mass fraction of cerium dioxide exhibited a relatively large overall variation and did not stabilize until after 3100 s. During the experiment, the minimum friction coefficient was 0.081, the maximum was 0.144, and the average friction coefficient was 0.108.

**Figure 2:**
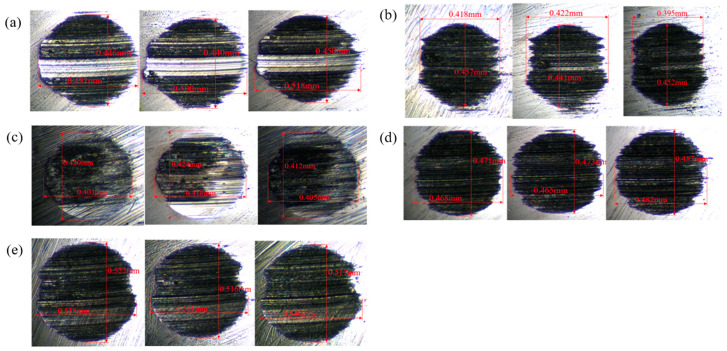
Wear scar diameter of polyurea-based gel greases with different CeO_2_ loadings. (**a**) Polyurea-based gel grease. (**b**) Polyurea-based gel grease + 0.1% CeO_2_. (**c**) Polyurea-based gel grease + 0.5% CeO_2_. (**d**) Polyurea-based gel grease + 1% CeO_2_. (**e**) Polyurea-based gel grease + 2% CeO_2_.

Figure 2 shows the scuff mark diameters of polyurea-based gel greases with different CeO_2_ loadings.

The wear marks on the steel balls treated with polyurea-based gel grease are shown in Figure 2a. The average wear scar diameters of the three balls, as observed under a microscope, were approximately 0.469 mm, 0.47 mm, and 0.484 mm, respectively; the average wear scar diameter for all balls was approximately 0.474 mm.

In polyurea-based gel grease systems, the polyurea thickener forms a three-dimensional network structure to entrap the mineral base oil. At the friction pair interface, the physical adsorption of base oil molecules onto the polyurea fibers results in only an extremely thin lubricating film. This film adheres to the metal surface via van der Waals forces and hydrogen bonds, and its shear strength and extreme pressure load-bearing capacity are both very limited. As the sliding process continues, the physically adsorbed film is easily squeezed out of the contact zone, while the three-dimensional network structure undergoes mechanical damage, leading to accelerated base oil loss. Consequently, micro-protrusions on the metal surfaces come into direct contact and are subjected to high stresses. Under these conditions, two primary wear mechanisms occur: first, micro-cutting, where hard micro-protrusions scrape the mating surface to form continuous grooves and generate slender wear particles; second, adhesive wear, where localized instantaneous high temperatures and pressures induce cold welding between micro-protrusions, followed by tearing under shear stress, resulting in spalling pits and material transfer. Since the system failed to generate any effective chemical reaction film or self-healing physical layer, the wear process was also accompanied by the initiation and propagation of micro-fatigue cracks. Consequently, the wear scar surface exhibited typical plow grooves, spalling pits, and fatigue striations, with an average wear scar diameter of approximately 0.474 mm. This wear morphology corresponds to the typical characteristics of unmodified grease under boundary lubrication conditions, indicating overall poor anti-wear performance. Therefore, further research into the grease composition and additives is required, along with strict control of experimental conditions, to ensure the stability and reliability of friction performance.

Figure 2b displays the wear marks on steel balls treated with polyurea-based gel grease containing 0.1% CeO_2_. After microscopic observation, the average wear scar diameters of the three balls were approximately 0.438 mm, 0.432 mm, and 0.424 mm, respectively, with the average wear scar diameter for all balls being approximately 0.431 mm.

Nano-cerium dioxide particles possess moderate hardness and a certain degree of chemical reactivity; during the initial stage of friction, they can enter the contact zone. Some of these particles form a cerium-containing physical adsorption film and a frictional chemical reaction film on the metal surface, while others roll between the friction pairs in an approximately spherical shape, converting part of the sliding friction into rolling friction, thereby reducing wear to a certain extent. However, due to the low dosage, although the particles are well dispersed and no significant agglomeration has occurred, the protective film that may form is likely to be thin and incomplete, resulting in direct contact between metal micro-protrusions in some areas; At the same time, the number of nanoparticles capable of participating in the rolling effect is limited, and the rolling lubrication effect is not fully realized. Therefore, while the anti-wear performance at a 0.1% addition level was superior to that of the original grease, the overall lubrication effect remained inadequate, necessitating further adjustment of the formulation.

Figure 2c displays the wear marks on steel balls treated with polyurea-based gel grease containing 0.5% CeO_2_. The average wear scar diameters of the three balls, as observed under a microscope, were approximately 0.412 mm, 0.421 mm, and 0.409 mm, respectively, with the average wear scar diameter for all balls being approximately 0.414 mm.

When the mass fraction of nano-cerium dioxide was increased from 0.1% to 0.5%, the wear scar diameter further decreased to approximately 0.414 mm. Nano-cerium dioxide particles form a uniform and dense dispersion system in the polyurea-based grease, primarily due to the moderate particle content and good compatibility with the grease matrix, effectively preventing particle agglomeration or sedimentation. The aforementioned performance improvements may be attributed to a synergistic protective mechanism, in which ultrafine particles continuously supplied by the grease accumulate on the friction surfaces and, through physical adsorption and potential frictional chemical reactions, initially form a load-bearing interfacial film, thereby suppressing direct contact between the metal micro-protrusions to some extent. However, it must be noted that direct XPS or Raman spectroscopic evidence regarding the precise chemical composition and bonding state of this friction film is currently lacking; the mechanism of “protective film formation” is presented here solely as a hypothesis awaiting verification. An appropriate amount of nanoparticles can embed themselves and fill the minute microgrooves already formed on the friction surfaces; under localized high temperature and pressure, they undergo sintering or compaction, thereby exerting a “self-healing” effect that smooths the worn surfaces. This ensures the reliable operation of drive motor bearings under high-temperature and high-speed conditions.

Figure 2d displays the wear marks on steel balls treated with polyurea-based gel grease containing 1% CeO_2_. The average wear scar diameters of the three balls, as observed under a microscope, were approximately 0.470 mm, 0.470 mm, and 0.485 mm, respectively, with the average diameter of all balls being approximately 0.474 mm.

When the CeO_2_ content was increased to 1%, the diameter of the wear scar increased significantly compared to the optimal value, and lubrication performance deteriorated. This phenomenon may be attributed to the difficulty of maintaining a uniform dispersion of excess CeO_2_ particles in the polyurea-based grease, leading to agglomeration in localized areas and the formation of micron-sized hard aggregates. During friction, when these aggregates enter the contact interface, they no longer function to form a friction film or to bear loads through particle deformation or embedding. Instead, they act as third-body abrasives, causing significant plowing and micro-cutting effects on the metal surface, thereby exacerbating wear. Excessive particle agglomeration may disrupt the continuity of the cerium-containing surface film formed at low concentrations, thereby reducing its protective effectiveness; at the same time, these accumulated particles may hinder the replenishment of base oil, leading to insufficient lubrication in the contact zone, which in turn can trigger a deterioration in boundary lubrication and even brief dry friction.

Figure 2e displays the wear marks on steel balls treated with polyurea-based gel grease containing 2% CeO_2_. The average scuff mark diameters of the three balls, as observed under a microscope, were approximately 0.530 mm, 0.524 mm, and 0.532 mm, respectively, while the average scuff mark diameter for all balls was approximately 0.528 mm.

When the CeO_2_ content was further increased to 2%, the scuff mark diameter reached its maximum value among all samples, indicating that the lubricating performance had deteriorated significantly. This phenomenon may be attributed to severe agglomeration of the particles within the grease, forming micron-sized hard aggregates that disrupted the original three-dimensional colloidal structure of the polyurea-based gel grease. This led to the separation of the base oil from the thickener, resulting in a significant decrease in the grease’s fluidity. Nanoparticles enter the contact zone in the form of agglomerates, embedding themselves in the metal substrate as hard abrasive particles, causing severe wear, a sharp rise in frictional heat, and accelerated oxidation and failure of the grease. As a result, scratches appear on the wear surface, and the anti-wear performance is significantly lower than that of the original grease.

#### Comparative Analysis of Tribology Experiments

Previous studies have typically focused on evaluating the performance of greases with a single concentration of nano-additives, or have simply assumed that higher addition levels result in better anti-wear performance. There has been a lack of systematic investigation into the effects of concentration gradients, and in particular, insufficient research into the mechanisms underlying performance degradation caused by excessive addition. This study reveals the concentration effects of nano-cerium dioxide in polyurea-based gel greases: moderate addition (0.5%) significantly enhances anti-wear performance, whereas further increases to 1% and 2% result in performance degradation, even falling below that of the base grease. Compared to the original grease (average wear scar diameter of 0.474 mm), the wear scar diameter decreased to 0.431 mm at a 0.1% loading, but the protective film was thin and the rolling effect was limited; at an addition level of 0.5%, the diameter of the scuff marks further decreased to 0.414 mm, reaching an optimal value. At this point, the nanoparticles formed a uniform and dense dispersion system, which may have generated a protective film at the friction interface and filled and repaired microgrooves, effectively suppressing micro-cutting and adhesive wear. When the addition level increases to 1%, the wear scar diameter rises back to 0.474 mm, as excess particles agglomerate to form micron-sized hard aggregates, triggering abrasive wear and destroying the protective film. When the addition level was further increased to 2%, the scuff mark diameter increased to 0.528 mm, and the anti-wear performance was the poorest. This may be due to the fact that high-concentration agglomerates disrupted the colloidal structure of the grease, reducing its fluidity and leading to severe abrasive wear and oxidative failure. Since all samples were processed using the same dispersion method, the performance differences are primarily attributed to CeO_2_ concentration rather than process variations; however, a decline in dispersion uniformity (local agglomeration) at high concentrations remains a possibility and requires further confirmation via SEM.

The statistical data in Table 1 indicate that the 0.5 wt% CeO_2_ sample had the lowest average coefficient of friction (0.086, SD ≤ 0.003, 95% CI [0.0794, 0.0926]), showing a significant difference compared to pure fat (0.096, [0.0917, 0.1003]), and the confidence intervals for higher concentrations (1% and 2%) shift upward overall, confirming that 0.5 wt% is the optimal addition level.

As shown in Table 2, the wear diameter first decreased and then increased with the addition of CeO_2_. The sample containing 0.5 wt% CeO_2_ exhibited the smallest average wear diameter (0.4137 mm, 95% CI [0.3975, 0.4299]), and compared with the pure fat (0.4742 mm, [0.4530, 0.4954]), the confidence intervals do not overlap and the difference is significant (*p* < 0.05). When the addition was increased to 2 wt%, the wear diameter significantly increased to 0.5283 mm, indicating that 0.5 wt% CeO_2_ is the optimal concentration.

**Figure 3:**
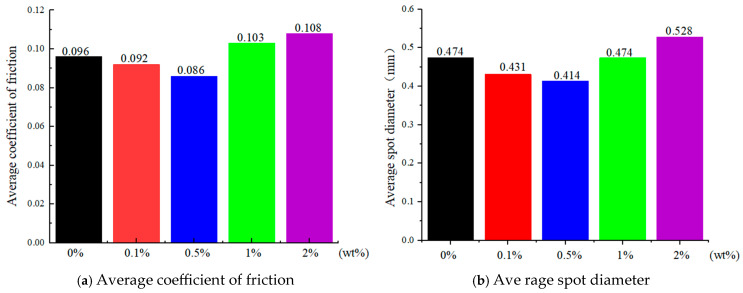
Average coefficient of friction and average wear scar diameter for the base grease and the experimental grease. (Black: Polyurea-Based Gel Grease. Red: Polyurea-Based Gel Grease with 0.1% CeO_2_. Blue: Polyurea-Based Gel Grease with 0.5% CeO_2_. Green: Polyurea-Based Gel Grease with 1% CeO_2_. Purple: Polyurea-Based Gel Grease with 2% CeO_2_).

Figure 3 presents the measured results for the average coefficient of friction and average wear scar diameter of the specimens at different additive levels, clearly revealing the trends in the anti-wear performance of polyurea-based gel grease with different concentrations of nano-cerium dioxide added. By comparing the average wear scar diameters, it can be clearly concluded that the polyurea-based gel grease with a 0.5% nano-cerium dioxide addition exhibits the smallest average wear scar diameter, indicating the best anti-wear performance.

Based on the experimental data, the appropriate addition of nano-cerium dioxide (0.5%) can significantly enhance the boundary lubrication capability of polyurea-based gel grease. For drive motors in new energy vehicles, this optimally formulated grease can effectively reduce wear on friction pairs, extend equipment service life under harsh operating conditions such as high temperatures and high speeds, and reduce maintenance frequency caused by wear-related failures.

### 2.2. Rheological Testing

#### 2.2.1. Shear Stress and Shear Rate

**Figure 4:**
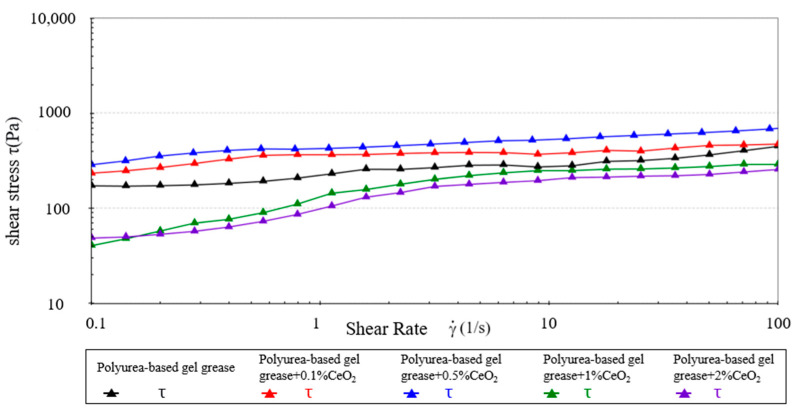
Shear stress–shear rate curves for base grease and blended grease.

Rheological tests were conducted on polyurea-based gel greases and their additive-containing formulations, yielding shear stress–shear rate curves (Figure 4).

High-temperature environments pose a serious threat to the physical and chemical stability of grease. Rising temperatures can significantly degrade grease performance, causing it to soften excessively and leak from lubricated areas in large quantities. This prevents the maintenance of a continuous lubricating film, leading to increased wear on the surfaces of friction pairs and ultimately resulting in equipment failure. By using grease with high shear stability, more consistent lubrication performance can be maintained in high-temperature environments. Under high-temperature and high-thermal-load conditions, the use of grease with high shear stability can significantly reduce grease loss caused by evaporation and migration, maintaining the thickness and continuity of the lubricating film, thereby effectively controlling wear. This extends the service life of drive motor bearings and enhances the reliability of system operation.

As shown in Figure 4, the initial shear stress of the polyurea-based gel grease is 173.25 Pa. After adding 0.5% by mass of nano-cerium dioxide, the initial shear stress increases to 283.66 Pa, indicating that this formulation exhibits significantly enhanced shear resistance under high-temperature conditions. These changes are primarily attributed to the following: at a 0.5% loading, the nanoparticles are uniformly dispersed in the grease and can participate in the formation of a stable and effective boundary lubrication film under high-temperature conditions, thereby suppressing grease loss under high-shear conditions and enhancing the overall shear resistance of the colloidal structure. At the same time, the introduction of nanoparticles helps stabilize the system’s viscosity, enabling the grease to maintain consistent rheological properties under various operating conditions.

However, when the additive content increased to 2%, the initial shear stress dropped to 49 Pa. This phenomenon is primarily attributed to the agglomeration effect of the nanoparticles: an excess of nanoparticles aggregates, disrupting the uniform and continuous three-dimensional network structure within the grease, leading to an abnormal increase in system fluidity and a decrease in lubrication efficiency. The agglomerates not only fail to exert the reinforcing effect that nanomaterials should inherently possess but also cause structural inhomogeneity and performance fluctuations, resulting in a significant decline in the grease’s overall performance.

The shear stress–shear rate curves were fitted using the Herschel–Bulkley model. The yield stress, consistency coefficient, and flow index obtained for different additive concentrations are shown in Table 3.

As shown in Table 3, when the additive content increased from 0% to 0.5%, the yield stress τ_0_ rose from 164.9 Pa to 289.3 Pa, indicating that an appropriate amount of additive enhanced the internal structural strength of the grease. At the same time, the flow index *n* increased from 0.367 to 0.462, indicating a slight decrease in shear thinning, but the grease remained a strongly shear-thinning fluid (*n* < 0.5). However, when the additive content was further increased to 1% and 2%, τ_0_ dropped sharply to near 0 Pa, and *n* rose to approximately 0.72, with the fluid behavior shifting from a Herschel–Bulkley type with yield stress to a power-law type without yield stress. This transition may be due to the excessive additive disrupting the grease’s colloidal network structure.

#### 2.2.2. Viscosity and Shear Rate

**Figure 5:**
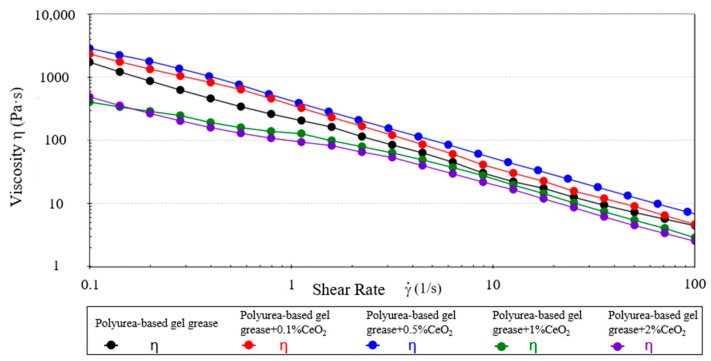
Viscosity-shear rate curves for base grease and blended grease.

Rheological tests were conducted on polyurea-based gel greases and their additive-containing formulations, yielding viscosity-shear rate curves (Figure 5).

Under high-temperature conditions, grease faces issues such as accelerated evaporation loss of base oil, softening of the thickener structure, and reduced stability of the colloidal system. This leads to easy leakage from the friction interface and a decrease in the load-carrying capacity of the lubricating film, which in turn causes abnormal equipment wear, increased operating resistance, and reduced reliability. Therefore, maintaining a high viscosity is key to enhancing the high-temperature performance of grease. A higher viscosity effectively slows down the volatilization and migration of the base oil, ensuring a continuous supply of lubricant to the contact zone. At the same time, high viscosity helps form and maintain a lubricating film with stronger dynamic load-bearing capacity, thereby directly reducing the wear rate. Selecting high-viscosity grease is a critical engineering strategy for addressing the risk of lubrication failure caused by high temperatures, extending the service life of bearings in new energy vehicle drive motors, and maintaining system operational stability.

As shown in Figure 5, the initial viscosity of the original grease was 1733 Pa·s. When the content of nano-cerium dioxide additive in the test grease was 0.5%. The initial viscosity increased to 2835 Pa·s, indicating significantly enhanced viscosity under high-temperature conditions. The high viscosity and non-Newtonian flow characteristics of polyurea-based gel greases stem from the three-dimensional fibrous network structure formed by the self-assembly of thickener molecules in the base oil. We hypothesize that when cerium dioxide particles are uniformly dispersed, they may penetrate and fill the voids within this network structure, with the particle surfaces interacting with the polyurea fibers through physical adsorption or electrostatic forces. This interaction appears to contribute to the formation of a denser and more robust internal structure; however, it should be noted that this inference is currently based solely on changes in apparent viscosity observed in steady-state flow tests and requires further validation through dynamic oscillation experiments. However, when the additive content was increased to 2%, the initial viscosity dropped to 489 Pa·s. This phenomenon is primarily due to the agglomeration effect of the nanoparticles, which disrupts the uniformity of the network, increases resistance to unstable flow, and hinders the formation of an effective friction film, ultimately leading to a deterioration in rheological performance.

By synergistically regulating the apparent viscosity and shear stress response of the grease, its steady-state flow characteristics can be improved to some extent, which may help enhance lubrication stability and adaptability to operating conditions. This improvement in flow characteristics may have a positive impact on maintaining the integrity of the lubricating oil film under high load and temperature conditions, thereby potentially leading to gains in the operational efficiency and reliability of mechanical systems. For bearings in new energy vehicle drive motors, the above findings suggest that such composite greases may have the potential to extend service life and reduce the risk of failure. However, it must be noted that this inference is currently based solely on the steady-state rheological and tribological experimental results of this study. Its effectiveness under actual high-temperature, high-speed operating conditions in motors still requires verification through dynamic rheological characterization and full-scale bench tests.

### 2.3. Microscopic Exploration

#### Analysis of Wear Surfaces

**Figure 6:**
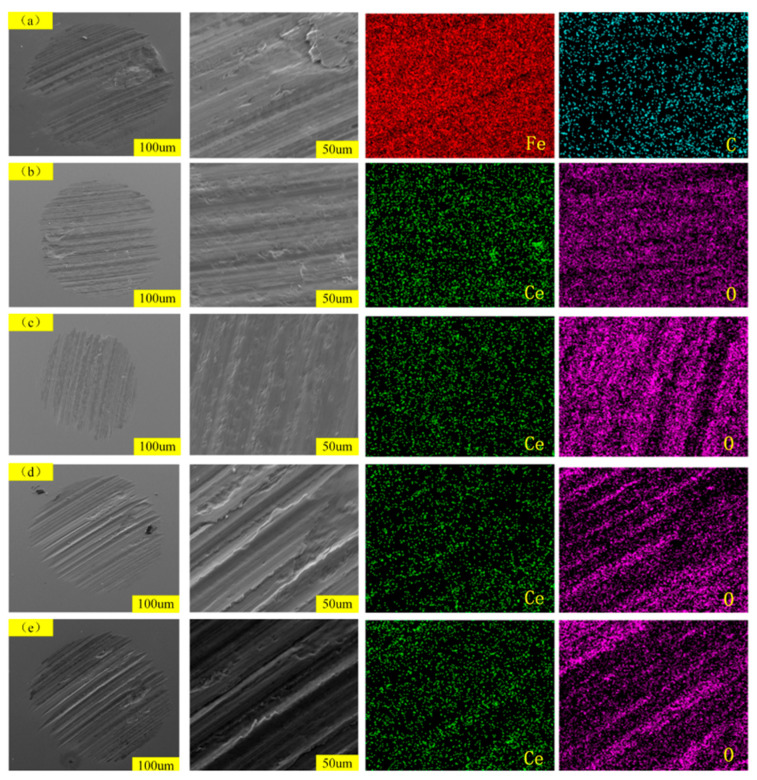
SEM micrographs and EDS elemental analysis. (**a**) Polyurea-based gel grease (**b**) Polyurea-based gel grease + 0.1% CeO_2_ (**c**) Polyurea-based gel grease + 0.5% CeO_2_ (**d**) Polyurea-based gel grease + 1% CeO_2_ (**e**) Polyurea-based gel grease + 2% CeO_2_.

A comparative analysis of the SEM microstructure and EDS elemental composition of steel ball wear marks under Polyurea-based gel grease with different CeO_2_ contents was conducted (Figure 6). The results indicate that there are significant differences in the protective effects of the five greases on the surfaces of the friction pairs, and the degree of wear exhibits a clear pattern as the additive content varies.

As shown in Figure 6, a comparison of SEM images of steel ball wear scars in polyurea-based gel greases with different CeO_2_ loadings indicates that the additive content has a significant effect on wear behavior. At a loading level of 0.5%, the abrasion grooves were shallowest and most evenly distributed, with no noticeable material spalling and the least wear. This suggests that an appropriate amount of CeO_2_ may form an effective protective film at the friction interface, significantly reducing abrasive and adhesive wear. At a 0.1% addition level, the wear scar characteristics were similar to those at 0.5%, with only a slight increase in groove depth. Although the wear severity was slightly higher, it remained superior to that of the pure base grease. Under lubrication with pure polyurea base grease, the wear grooves were more pronounced, with minor surface damage observed. Lacking the reinforcing effect of additives, its anti-wear performance was limited. When the CeO_2_ content was increased to 1% and 2%, the wear grooves became significantly deeper and wider, accompanied by extensive material spalling and plastic deformation. Particularly at a 2% content, particle agglomeration intensified, and the three-body abrasive wear effect increased, leading to reduced lubricant film stability and markedly worsened wear severity. Overall, the CeO_2_ additive exhibits a “first improvement, then deterioration” trend in the anti-wear performance of polyurea grease. The optimal addition ratio is 0.5%; excessive addition exacerbates wear due to particle agglomeration, thereby reducing the lubricating effect.

The surface elemental composition of the samples at different Ce loading levels is shown in Table 4.

EDS analysis indicates that the speciation of Ce changes significantly as the additive concentration increases. At 0.5%, the Ce content is 4.6 wt% and the O content is only 7.5 wt%; Ce is loaded in a highly dispersed and active form, resulting in optimal performance. Below this concentration, there is an insufficient number of active sites; above this concentration, two failure modes occur: at 1%, the Ce content drops abnormally while the C content rises abnormally, indicating agglomeration of Ce species; at 2%, although the total Ce content reaches its maximum, the O content surges sharply, indicating the formation of a large amount of inactive CeO_2_ phase, which covers the active sites and disrupts the structure. In summary, 0.5% is the optimal Ce doping concentration, achieving the best balance between high dispersion of active species and low oxidative side reactions.

**Figure 7:**
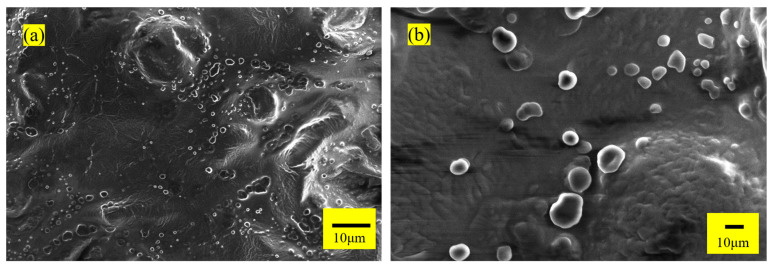
SEM micrographs of the polyurea-based gel grease containing 2 wt% CeO_2_ at different magnifications: (**a**) Accelerating voltage: 15 kV, magnification: ×200 (**b**) Accelerating voltage: 15 kV, magnification: ×1000.

Figure 7 shows the SEM images of Polyurea-based gel grease and a sample containing 2 wt% CeO_2_.

As shown in the figure, when the additive concentration reaches 2%, significant agglomeration of CeO_2_ particles occurs within the polyurea soap fiber network. A large number of fine particles aggregate to form agglomerates of varying sizes, resulting in a non-uniform distribution. At the same time, the originally continuous three-dimensional polyurea soap fiber network is compromised; in some areas, the intertwined fibers are stretched apart and broken, disrupting the integrity of the network. This agglomeration and structural degradation weaken the grease’s colloidal stability and oil-retention capacity. Furthermore, large agglomerates tend to act as hard abrasive particles during friction, exacerbating abrasive wear on the friction pair. This is consistent with the severe wear observed in the wear scar morphology at a 2% additive concentration, indicating that excessive CeO_2_ addition disrupts the grease structure and, conversely, degrades its anti-wear performance.

## 3. Conclusions

This study systematically investigated the effects of nano-cerium dioxide (at concentrations ranging from 0 to 2 wt%) on the tribological and rheological properties of polyurea-based gel greases. The main findings are as follows.

The addition of 0.5 wt% nano-cerium dioxide yields the best overall performance. Compared to the base grease, the average coefficient of friction decreased by 10.4% (from 0.096 to 0.086), and the average wear scar diameter decreased by 12.7% (from 0.474 mm to 0.414 mm). Compared to the sample containing 0.1 wt% nano-cerium dioxide, the average coefficient of friction decreased by 6.98% (from 0.092 to 0.086), and the average scuff mark diameter decreased by 4.11% (from 0.431 mm to 0.414 mm). Compared to the sample containing 1 wt% nano-cerium dioxide, the average coefficient of friction decreased by 19.8% (from 0.103 to 0.086), and the average wear scar diameter decreased by 14.5% (from 0.474 mm to 0.414 mm). Compared to the addition of 2 wt% nano-cerium dioxide, the average coefficient of friction decreased by 25.6% (from 0.108 to 0.086), and the average scuff mark diameter decreased by 27.5% (from 0.528 mm to 0.414 mm); furthermore, the coefficient of friction exhibited the least fluctuation, making it the optimal choice among the tested concentrations. Concurrently, the initial shear stress of the 0.5 wt% formulation grease increased from 173.25 Pa to 283.66 Pa, a rise of approximately 63.7%; its viscosity increased from 1733 Pa·s to 2835 Pa·s, a rise of approximately 63.6%. The results of the rheological fitting indicate that the yield stress of the grease is highest (289.3 Pa) at an additive concentration of 0.5%; as the concentration increases to 1% and beyond, the yield stress approaches zero, and the system transforms into a shear-thinning fluid with no yield stress. These performance improvements stem from two synergistic mechanisms: well-dispersed nanoparticles fill surface microdefects at the sliding interface through deformation and embedding, and participate in frictional chemical reactions to form a protective friction film, thereby reducing friction and wear; simultaneously, nanoparticles may interact with the polyurea fiber network through electrostatic and physical adsorption, thereby enhancing the apparent strength of the gel’s three-dimensional network structure and improving its resistance to shear thinning. It should be noted that direct confirmation of this interaction requires spectroscopic evidence, such as FTIR or XPS analysis, which has not yet been conducted in this study but could be further verified in future work.

The value of this study lies in demonstrating that, at an optimized concentration, nanoparticles can simultaneously improve the anti-friction, anti-wear, and shear resistance properties of polyurea grease. The enhanced viscosity retention capability at higher shear rates is particularly important for drive motor bearings that must operate over a wide range of speeds and temperatures—maintaining a stable lubricating film is key to preventing boundary lubrication failure. Nevertheless, more accurate confirmation of the network structure still requires characterization methods such as dynamic oscillation testing.

However, this study also has limitations. First, all tribological tests were conducted under fixed load, speed, and temperature conditions, failing to account for the effects of dynamic variations in actual operating conditions; the optimal loading of nano-CeO_2_ may shift depending on operating conditions. Second, only short-term tribological performance was evaluated, and the long-term colloidal storage stability and agglomeration behavior of the nanoparticles in the grease were not systematically investigated.

Based on the above findings, future work should focus on the following areas: Systematically evaluate the tribological response of this ultrafine CeO_2_ grease formulation across a wide range of loads, sliding speeds, and ambient temperatures to determine its applicable limits. Conduct long-term storage stability studies using rheological characterization, dynamic light scattering, and electron microscopy to reveal the dispersion state and evolution patterns of particles during prolonged storage and use, and supplement these with ATR-FTIR spectra of the grease before and after testing to confirm the chemical stability of the base grease. Systematically evaluate the effects of different CeO_2_ loading levels on the thermal stability and high-temperature behavior of the composite grease through differential scanning calorimetry (DSC), thermogravimetric analysis (TGA), and drop point testing, thereby providing a basis for thermal performance in high-temperature motor bearing applications.

Furthermore, this study conducted only a preliminary screening within a relatively broad concentration range of 0.1–2.0 wt%, which is insufficient to precisely identify the performance inflection point. Future work could establish a more refined concentration gradient between 0.5 wt% and 1 wt% to systematically investigate the evolution of the grease’s tribological and rheological properties within this range, thereby determining whether an optimal concentration exists and providing a more precise formulation basis for the engineering applications of this system. For the initial screening, mild operating conditions were selected. In actual operation, the drive motor is subjected to high temperatures exceeding 120 °C and high rotational speeds exceeding 10,000 rpm, which introduce new challenges such as centrifugal forces and thermal degradation. The actual performance of this formulation must be verified through more rigorous full-scale bearing bench tests.

## 4. Materials and Methods

### 4.1. Selection of Greases and Additives

**Figure 8:**
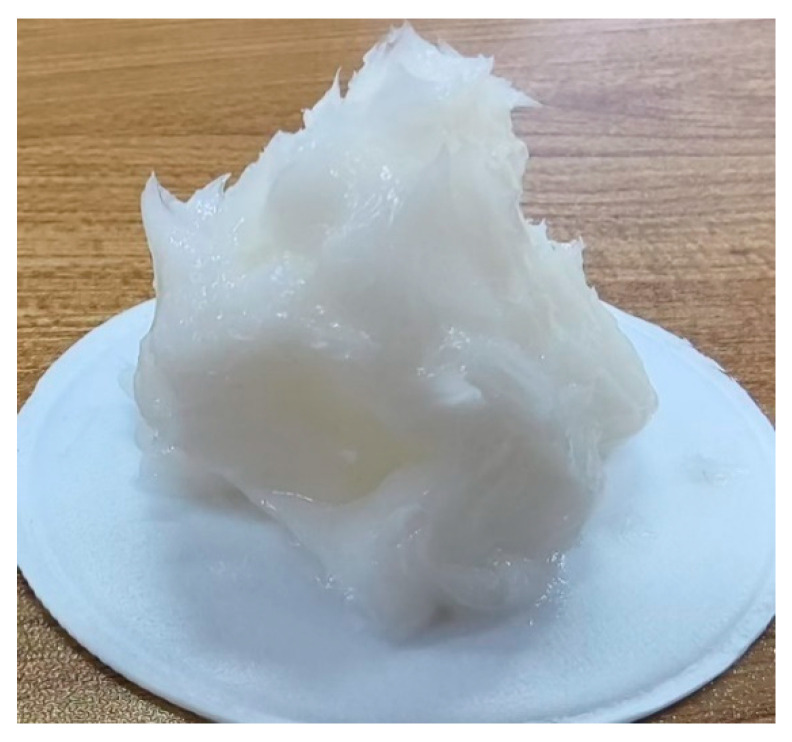
Polyurea-based gel grease.

The grease used in this experiment is a polyurea-based gel grease (Figure 8), supplied by Guangzhou Fuxi Lubrication Technology Co., Ltd., Suzhou, China The relevant parameters are shown in Table 5. Polyurea-based gel grease is a thermoreversible physical gel that uses organic urea compounds as gelling agents and forms a three-dimensional network structure through intermolecular hydrogen bonding. As a high-performance gel lubricant, it is renowned as a “long-life grease” due to its exceptional comprehensive performance. The core advantage of this gel system lies in its excellent high-temperature resistance and oxidation stability, enabling it to maintain the integrity of its gel network structure over extended periods at high temperatures without coking or hardening, resulting in a service life far exceeding that of traditional lithium-based greases. Additionally, its three-dimensional gel framework endows the material with excellent mechanical stability, water resistance, and corrosion resistance, along with good compatibility with various sealing materials. Polyurea-based gel greases are widely used in applications with demanding lubrication requirements, primarily including high-temperature environments (such as motor bearings), long-life maintenance-free equipment, and humid or special operating conditions. By extending maintenance intervals and enhancing equipment reliability, this gel-type grease serves as an efficient lubrication solution that effectively reduces total costs.

**Figure 9:**
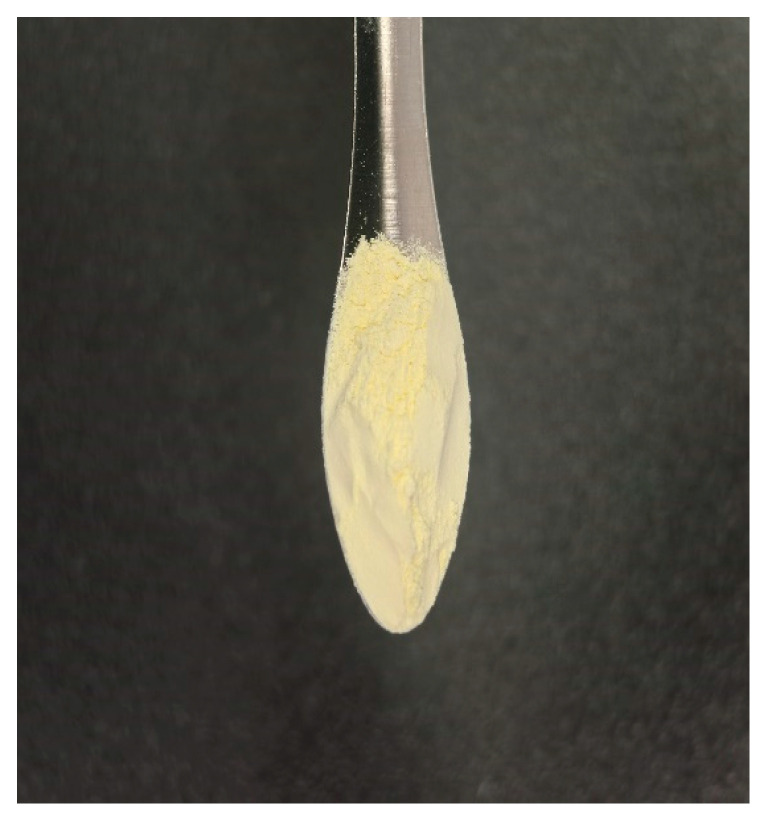
Nano-cerium dioxide.

The additive used in this experiment is cerium dioxide (CeO_2_) with a particle size of 50 nm (Figure 9), provided by Suzhou Youyan New Materials Industrial Co., Ltd., Suzhou, China Cerium dioxide is a rare earth oxide with exceptional performance. Its core value stems from its unique fluorite crystal structure. Its excellent oxygen storage and release capabilities allow it to act as a “chemical oxygen buffer” by dynamically forming oxygen vacancies—a mechanism that underpins its highly efficient catalytic performance. It plays a key role in the field of energy catalysis. Furthermore, it possesses good ionic conductivity, high-temperature stability, UV absorption capabilities, and biocompatibility, offering broad application prospects.

This study reports only the dropping point data (250 °C) for the polyurea-based gel grease; the effects of different CeO_2_ loading levels on the dropping point and thermal stability of the composite grease have not been systematically investigated in this work. Given that thermal stability is a key performance indicator for high-temperature motor bearing greases, this aspect requires further evaluation in future studies.

### 4.2. Forms of Additives

**Figure 10:**
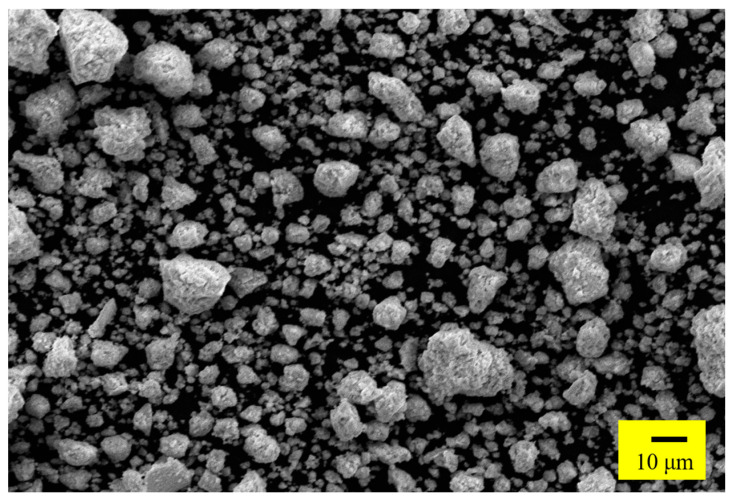
Microstructure of cerium dioxide (accelerating voltage: 15 kV, magnification: ×1000).

The microstructure of the CeO_2_ powder was characterized using a scanning electron microscope (SEM) (Figure S3) under the following conditions: an acceleration voltage of 15 kV, a working distance of 14.7 mm, and high-vacuum secondary electron mode. As shown in Figure 10, the CeO_2_ particles are generally spherical in shape and exist as loose, soft agglomerates. The primary particle size distribution ranges from 1 to 5 μm, while the larger particles formed by soft agglomeration are mostly between 5 and 20 μm, with some reaching 20 to 30 μm. The particles are distributed relatively uniformly within the field of view. This micron-scale spherical morphology and moderate soft agglomeration are conducive to uniform dispersion in polyurea grease, making it suitable for use as a grease additive.

### 4.3. The Microstructure of Grease

**Figure 11:**
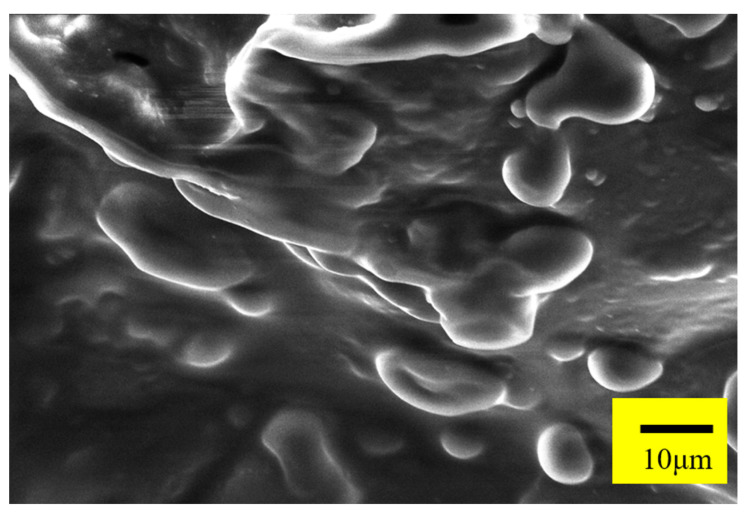
Microstructure of polyurea-based gel grease (accelerating voltage: 15 kV, magnification: ×2000).

Scanning electron microscopy (SEM) observations of polyurea-based gel grease (Figure 11).

SEM micrographs of polyurea-based gel grease reveal a continuous, three-dimensional, interwoven network of polyurea soap fibers within the grease. These soap fibers overlap and intertwine to form a stable framework; the base oil is encapsulated within the pores of the fiber network, creating a soap fiber–base oil colloidal composite system. Overall, the polyurea soap fibers exhibit a regular morphology and smooth surface, with an intact network structure showing no obvious fiber breakage, agglomeration, or structural collapse. This indicates that pure polyurea grease possesses excellent colloidal stability and mechanical stability, providing a structural foundation for the stable storage of the base oil and the subsequent formation of a lubricating film.

### 4.4. Experimental Design

#### 4.4.1. Sample Preparation

In this study, a series of CeO_2_ nanocomposite greases with mass fractions of 0, 0.1, 0.5, 1, and 2 wt% were prepared using a polyurea-based gel grease as the base material; 40 g of each sample was prepared. Specifically, the 0 wt% sample consisted of 40 g of base grease without any additives; for the 0.1 wt% sample, 0.04 g of nano-CeO_2_ was added to 39.96 g of base grease; for the 0.5 wt% sample, 0.2 g of nano-CeO_2_ was added to 39.8 g of base grease; for the 1 wt% sample, 0.4 g of nano-CeO_2_ was added to 39.6 g of base grease; for the 2 wt% sample, 0.8 g of nano-CeO_2_ was added to 39.2 g of base grease. The specific preparation steps are as follows: Ultrafine cerium dioxide powder with an average particle size of 50 nm and a purity of 99.9% was gradually added in batches to the polyurea-based gel lubricant at mass fractions of 0.1 wt%, 0.5 wt%, 1 wt%, and 2 wt% at room temperature (25 ± 2 °C). To achieve the most uniform mixing possible, manual mixing was performed for approximately 15 min using a stainless steel spatula through repeated folding, kneading, and circular stirring. During this process, the mixing was paused every 5 min to completely scrape off the material adhering to the container walls and the spatula and reincorporate it into the mixture until the color appeared visually uniform. Pure polyurea-based gel grease underwent the same mixing procedure as a control. All samples were sealed and stored after preparation. It should be noted that the dispersion method used in this study was manual mechanical stirring, and the quality of dispersion was indirectly assessed based on visual uniformity and the reproducibility of test results; microscopic techniques have not yet been employed for quantitative characterization of particle distribution. Future studies will consider incorporating processes such as three-roll milling or ultrasonic-assisted dispersion and will conduct quantitative evaluations of the dispersion effectiveness.

#### 4.4.2. Design of Tribology Experiments

In this study, an MS-10A four-ball friction (Xiamen Tianji Automation Co., Ltd., Xiamen, China) (Figure S1) and wear tester was used to investigate the tribological properties of virgin grease and modified grease. In the experiments, 12.7-mm-diameter steel balls were made of GCr15 bearing steel (AISI-52100) (Xiamen Tianji Automation Co., Ltd., Xiamen, China) (66 HRC). First, add approximately 6 g of test grease to the bottom of the grease cup so that the three stationary steel balls to be added later are completely submerged. After adding the steel balls, add another 4 g of grease to ensure that the contact points between the rotating steel ball and the three stationary steel balls are completely submerged in the grease. Before and after each test, all relevant machine components (including the upper and lower bases, the grease cup, and the new test balls) were cleaned in an ultrasonic cleaner containing petroleum ether. The coefficient of friction was automatically recorded by the computer connected to the friction tester. Three four-ball tests were conducted under identical conditions to ensure repeatability. The results presented in this paper are the average of these three tests. The equipment test conditions are summarized in Table 6.

#### 4.4.3. Design of Rheological Experiments

The MCR rotational rheometer (Figure S2) used in this study is a comprehensive, advanced analytical instrument designed to characterize the rheological properties of various materials. This instrument can accurately measure multiple key rheological parameters, including shear stress, shear rate, and viscosity, thereby providing quantitative data for evaluating the flow and deformation behavior of materials under applied forces. It features a wide range of technical specifications, with a rotatable speed adjustable between 10^−6^ and 200 r/min, a torque measurement range of 10 μN·m to 0.2 N·m, and support for normal force loading from 0.001 to 50 N. The instrument integrates the RheoCompass intelligent rheology software version 1.25 (Anton Paar (China) Co., Ltd., Shanghai, China), which employs a modular architecture to standardize the entire testing and analysis workflow. It combines functions such as experimental design, real-time data and graphical display, advanced analysis, and report generation. The core advantage of this software lies in its ability to handle complex rheological models and to predict and simulate material rheological behavior based on test data. The test conditions for the equipment are listed in Table 7.

The rheological data were fitted nonlinearly using the Herschel–Bulkley model, with the following equation:

$$\tau \;=\;\tau_{0}\;+\;k{\left(\dot{\gamma }\right)}^{n}$$

where *τ* is the shear stress, $\dot{\gamma }$ is the shear rate, $\tau_{0}\;$is the yield stress, k is the consistency coefficient, and *n* is the flow index. The shear stress and shear rate data for the five samples collected during the experiment were imported into Origin 2021 software. A fitting program was written, and the experimental data were imported into the program. The fitting program was run to perform parameter fitting for the H-B model, yielding the parameters $\tau_{0}$, k, and n.

## Figures and Tables

**Table 1:** Average friction coefficients ± standard deviation and 95% confidence intervals for different CeO_2_ loading levels.

Sample	Average	Standard Deviation (SD)	95% Confidence Interval
1	0.096	0.0017	[0.0917, 0.1003]
2	0.092	0.0027	[0.0854, 0.0986]
3	0.086	0.0027	[0.0794, 0.0926]
4	0.103	0.0030	[0.0956, 0.1104]
5	0.108	0.0027	[0.1014, 0.1146]

Note: The confidence interval is based on the t-distribution (degrees of freedom = 2, t_0.025,2_ = 4.303), and is calculated as the mean ± (t × standard error). Sample 1 = Polyurea-Based Gel Grease, Sample 2 = Polyurea-Based Gel Grease with 0.1% CeO_2_, Sample 3 = Polyurea-Based Gel Grease with 0.5% CeO_2_, Sample 4 = Polyurea-Based Gel Grease with 1% CeO_2_, Sample 5 = Polyurea-Based Gel Grease with 2% CeO_2_.

**Table 2:** Average Wear scar diameter ± Standard Deviation and 95% Confidence Interval for Different CeO_2_ Loading Levels.

Sample	Average	Standard Deviation (SD)	95% Confidence Interval
1	0.474	0.00855	[0.4530, 0.4954]
2	0.431	0.00702	[0.4134, 0.4482]
3	0.414	0.00653	[0.3975, 0.4299]
4	0.474	0.00881	[0.4524, 0.4962]
5	0.528	0.00426	[0.5177, 0.5389]

Note: Degrees of freedom = 2, t_0.025,2_ = 4.303. The formula for the confidence interval is: mean ± (t × standard error), where standard error = SD/√3. Sample 1 = Polyurea-Based Gel Grease, Sample 2 = Polyurea-Based Gel Grease with 0.1% CeO_2_, Sample 3 = Polyurea-Based Gel Grease with 0.5% CeO_2_, Sample 4 = Polyurea-Based Gel Grease with 1% CeO_2_, Sample 5 = Polyurea-Based Gel Grease with 2% CeO_2_.

**Table 3:** Herschel–Bulkley fitting parameters for greases with different additive contents.

Additive Content (%)	Yield Stress τ_0_ (Pa)	Consistency Coefficient K (Pa·s^2^)	Flow Index *n*
0	164.9	45.28	0.367
0.1	236.1	32.53	0.444
0.5	289.3	31.74	0.462
1	0	51.06	0.715
2	0	52.78	0.703

Note: The yield stresses for concentrations of 1% and 2% are extremely low (the fitted values approach zero), so these materials can be considered power-law fluids with no yield stress.

**Table 4:** EDS Elemental Composition of Samples at Different Additive Concentrations.

Additive Concentration	C (wt%)	O (wt%)	Ce (wt%)
0%	8.5	6.9	0
0.1%	8.7	7.0	3.9
0.5%	5.2	7.5	4.6
1%	13.1	11.1	2.7
2%	10.4	21.1	5.4

**Table 5:** Specifications for Polyurea-Based Gel Grease.

Grease	Base Oil Viscosity	NLGI Consistency Grade	Operating Temperature	Dropping Point	Corrosion Resistance	Oxidation Resistance
Polyurea-based Gel Grease	Mineral oil 120 mm^2^/s. (at 40 °C)	2	−20~160 °C	250 °C	High	High

**Table 6:** Test Conditions.

Test Method	Test Parameters
Equipment Model	MS-10A Four-Ball Friction and Wear Tester
Spindle Speed	1200 r/min
Temperature	80 °C
Load	392 N
Time	3600 s

**Table 7:** Test Conditions.

Test Method	Test Parameters
Equipment Model	MCR Rotational Rheometer
Shear Rate Range	0.01~100 s^−1^
Shear Strain Range	0.1~100%
Temperature	80 °C
Time	15 s

## Data Availability

The data presented in this study are openly available in article.

## Supplementary Materials

The following supporting information can be downloaded at https://www.mdpi.com/article/10.3390/gels12060528/s1, Figure S1: MS-10A Four-Ball Friction and Wear Tester; Figure S2: MCR 302 Rotational Rheometer; Figure S3: Scanning Electron Microscope.
