# Supplementary material for: Study on the Properties of Nano-CeO2/Polyurea-Based Gel Grease for Electric Motor Bearings
Source: Gels. 2026 Jun 12;12(6):528. doi: 10.3390/gels12060528 (PMC13299449; doi:10.3390/gels12060528)
Supplement: Supplementary file 1 [file gels-12-00528-s001.zip › gels-4337165-supplementary.pdf]

1.1. *Laboratory Equipment*

1.1.1. Inertial Friction and Wear Tester

The MS-10A Four-Ball Friction and Wear Tester (Figure 1) is an advanced laboratory testing device manufactured by Xiamen Tianji Automation Co., Ltd., widely used for evaluating the performance of greases. This friction and wear tester is driven by a 2 kW servo motor, with a spindle speed adjustable within the range of 50 to 3000 r/min, an axial loading range of 49 to 9800 N, and a maximum oil cup heating temperature of 200 °C. Based on the classic four-ball friction test principle, this equipment simulates the actual sliding friction process between mechanical components, collects real-time data such as friction force and friction coefficient, and thereby quantitatively evaluates the load-carrying capacity and anti-wear performance of greases. In addition to testing the mechanical properties of materials, this equipment can be used in conjunction with the TDS-0745-MV microscope to observe the wear morphology on the steel ball surface and the diameter of wear pits. With a measurement accuracy of 0.001 mm for wear pits, it facilitates further investigation of material wear behavior and evaluation of wear resistance.

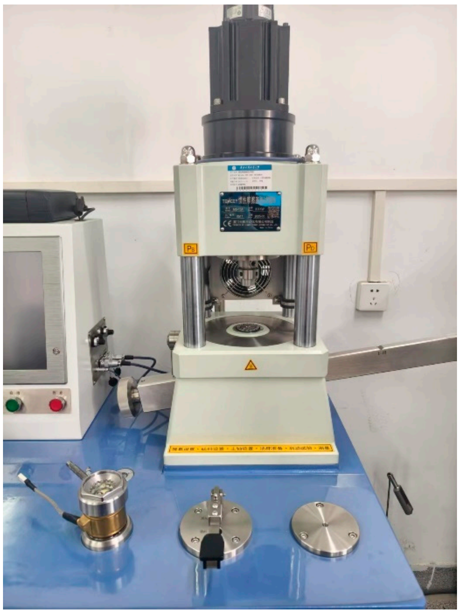

Figure S1. MS-10A Four-Ball Friction and Wear Tester.

1.1.2. MCR Rotational Rheometer

The Anton Paar MCR302 Rotational Rheometer is a laboratory instrument designed based on the principle of rotational shear (Figure 2). The instrument's rotational speed range spans 10^{-6} to 200 r/min, with a torque measurement range of 10 μN·m to 0.2 N·m, and it features precise normal force control from 0.001 N to 50 N. Its core drive system utilizes EC motors and air bearing technology, combined with high-

sensitivity torque sensors and optical encoders, enabling the accurate execution of various rheological test modes, including steady-state shear, dynamic oscillation, and transient testing. This allows for the comprehensive characterization of key rheological parameters of grease, such as steady-state flow curves, yield stress, thixotropy, and viscoelastic modulus. It provides a reliable and flexible experimental platform for in-depth research into the rheological behavior of grease under complex operating conditions.

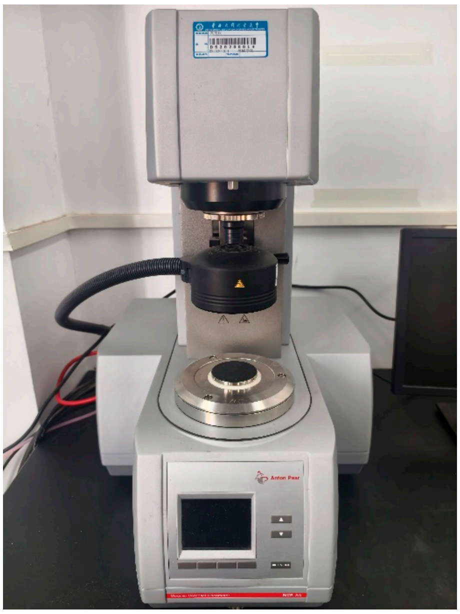

Figure S2. MCR 302 Rotational Rheometer.

1.1.3 Scanning Electron Microscope

The EM-30AXN is a benchtop scanning electron microscope developed by Korea's KUSEM (Figure 3), featuring a compact design, user-friendly operation, and cost-effectiveness. The instrument utilizes a pre-aligned tungsten filament electron source with an acceleration voltage continuously adjustable from 1 kV to 30 kV. It offers a magnification range of 15x to 150,000x, and at an acceleration voltage of 30 kV, its secondary electron image resolution is better than 5 nm. The instrument comes standard with a secondary electron detector and a backscattered electron detector, enabling surface analysis (SEM) and energy dispersive spectroscopy (EDS) of steel ball wear marks to compare the morphology and surface roughness of different samples, thereby meeting diverse analytical needs.

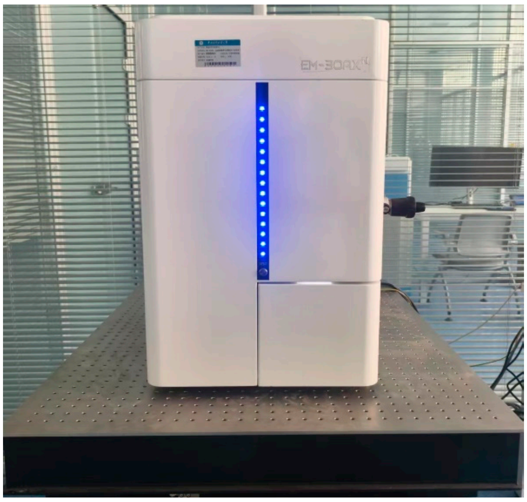

Figure S3. Scanning Electron Microscope.
